# More than Rehabilitation: The Role of Contextual Factors in Pain Perception and Recovery: What the Physiotherapy Literature Reports—A Narrative Review with a Systematic Search Strategy

**DOI:** 10.3390/jcm15166489

**Published:** 2026-08-21

**Authors:** Giulia Leonardi, Francesco Bonanno, Angelo Alito, Francesca Cucinotta, Clara Lombardo, Antonio Di Dio, Francesca Sposito, Carmen Cucinotta, Alfio Garofalo, Simona Portaro

**Affiliations:** 1 Department of Physical and Rehabilitation Medicine, University Hospital “G. Martino”, 98125 Messina, Italy; giulia.leonardi@polime.it (G.L.); simonaportaro@hotmail.it (S.P.); 2 Department of Clinical and Experimental Medicine, University of Messina, 98125 Messina, Italy; ciccio.bonanno@yahoo.it (F.B.); antoniodidio23@yahoo.com (A.D.D.); francescasp568@gmail.com (F.S.); cucinottacarmen181@gmail.com (C.C.); garofaloalfio8@gmail.com (A.G.); 3 Department of Biomedical, Dental Sciences and Morphological and Functional Images, University Hospital “G. Martino”, 98124 Messina, Italy; alitoa@unime.it; 4 IRCCS Centro Neurolesi Bonino-Pulejo, S.S. 113 Via Palermo, C. da Casazza, 98124 Messina, Italy; 5 Department of “Scienze della Salute”, University of “Magna Graecia”, 88100 Catanzaro, Italy; clara.lombardo@unicz.it

**Keywords:** contextual factors, musculoskeletal pain, non-verbal communication, patient–physiotherapist relationship, physiotherapy, placebo effect, rehabilitation

## Abstract

**Background**: Pain is a multidimensional experience shaped not only by nociceptive input but also by cognitive, emotional, and social processes. In musculoskeletal (MSK) rehabilitation, outcome variability among patients with similar clinical presentations suggests that determinants beyond tissue pathology may influence pain perception and recovery. Contextual factors (CFs)—communication, therapeutic relationship, patient expectations, and environmental cues—are increasingly recognized yet remain inconsistently integrated into routine practice. **Objective**: To map how CFs are represented in the physiotherapy literature indexed under that terminology, and to identify where empirical evidence in MSK rehabilitation is present and where it is absent. **Methods**: A narrative review with a systematic search strategy, reported following SANRA, was conducted in PubMed and Scopus, searched on 31 March 2026. Records were screened against predefined eligibility criteria, including publication from 2006 onwards and English language. Two reviewers independently screened records and resolved disagreements by consensus. Methodological quality was appraised using the Cochrane RoB2 tool for randomized controlled trials and an adapted Newcastle–Ottawa Scale for observational and cross-sectional studies. Conceptual publications were not formally appraised. Each publication was classified as direct clinical evidence (Tier A), indirect mechanistic or implementation-level evidence (Tier B), or conceptual contribution (Tier C). **Results**: Eight publications were included: no source provided direct clinical evidence in a musculoskeletal rehabilitation population (Tier A), five provided indirect mechanistic or implementation-level evidence (Tier B), and three were conceptual contributions (Tier C). The single randomised controlled trial retrieved was conducted in asymptomatic volunteers and was at high risk of bias for the between-group comparisons of all reported outcomes. Findings are organised as contextual domains, mediating mechanisms, and outcomes. No direct clinical evidence was retrieved; claims regarding expectations, therapeutic alliance, non-verbal communication, and the clinical environment rest on indirect or conceptual sources. **Conclusions**: CFs may contribute to pain and rehabilitation variability through cognitive, emotional, and relational pathways, complementing rather than replacing evidence-based care. The scarcity of direct clinical evidence within the literature indexed under this terminology is itself a finding. Construct-level searches and pragmatic trials are required to clarify the contribution of individual contextual domains.

## 1. Introduction

The International Association for the Study of Pain (IASP) defines pain as an ‘unpleasant sensory and emotional experience’ associated with actual or potential tissue damage [1]. Therefore, pain is not merely a biological signal, but a multidimensional experience influenced by sensory input, emotions, expectations, and social context [2]. This perspective emphasises the fundamental principle that pain emerges from the ongoing interaction between body, mind and environment [3].

In clinical practice, pain is typically categorised by duration and underlying mechanisms. Acute pain serves a protective function by supporting tissue healing [4], whereas chronic pain persists for more than three to six months [5].

From a mechanistic perspective, pain may arise from nociceptive, inflammatory, neuropathic or nociplastic processes, which may occur in combination [6]. However, this classification alone does not fully explain the substantial variability observed in pain experienced by patients with similar injuries or diagnoses.

People with similar tissue damage may report very different levels of pain intensity, disability, psychological distress, treatment response and recovery times. Likewise, rehabilitation outcomes may differ substantially despite the application of similar therapeutic interventions. Such observations suggest that recovery is influenced not only by biological factors but also by a broad range of cognitive, emotional, relational, and environmental determinants acting throughout the rehabilitation process [7,8,9].

Within this framework, increasing attention has been directed toward contextual factors (CFs). According to the International Classification of Functioning, Disability and Health, CFs encompass the personal and environmental circumstances surrounding an individual’s health condition [10]. More specifically, within rehabilitation, CFs have been described as the internal and external elements characterizing the therapeutic encounter that can influence patients’ expectations, perceptions, treatment responses, and clinical outcomes independently of the specific therapeutic intervention itself [11,12,13,14].

Recognising and actively managing CFs may help clinicians improve therapeutic outcomes and deliver more meaningful and personalised care. However, evidence on CFs has been scattered across different disciplines, and their clinical relevance in rehabilitation has often been poorly recognised or applied inconsistently [15,16] or secondary to an identifiable pathology [17]. Therefore, the same treatment may have different outcomes depending on how it is presented, communicated, and delivered within the clinical context.

This growing body of evidence has become increasingly prevalent in physiotherapy practice. Building upon this conceptualization, Testa and Rossettini have proposed a comprehensive framework in which CFs are organized into five major domains: patient-related factors, practitioner-related factors, the therapeutic relationship, treatment characteristics, and the clinical environment [12]. These domains influence patients’ perceptions of the intervention and contribute to its overall effectiveness.

Similarly, Cook and colleagues developed an international consensus taxonomy further supporting this multidimensional perspective and emphasizing the importance of contextual influences across different rehabilitation environments [13]. Together, these models suggest that CFs should not be regarded as incidental aspects of care but rather as integral components of the therapeutic process.

Among the most extensively investigated contextual mechanisms are patient expectations, placebo and nocebo responses, verbal and non-verbal communication, clinician empathy, therapeutic alliance, and characteristics of the clinical environment [12,13,18,19,20,21,22]. Positive expectations, effective communication, and supportive therapeutic relationships have been associated with improved patient engagement, greater treatment adherence, enhanced self-efficacy, and more favorable patient-reported outcomes [23,24,25,26,27]. Conversely, negative communication, maladaptive expectations, or threatening environmental cues may contribute to increased pain perception, fear, avoidance behaviors, and poorer rehabilitation experiences through nocebo-related mechanisms [22,28,29].

Environmental cues, such as lighting, noise levels, and the organisation of the clinical setting, may convey signals of safety or threat, thereby facilitating or hindering recovery processes [26].

Integrating these contextual dimensions appears to align rehabilitation practice with a biopsychosocial approach, emphasising that effective care may depend not only on the treatment provided, but also on how it is delivered.

Despite the growing interest in this area, evidence on CFs is fragmented across different study designs and disciplines. It is rarely appraised for methodological quality and has rarely been synthesized specifically for musculoskeletal rehabilitation. This leaves clinicians without a critical, rehabilitation-focused overview of how these factors influence pain and recovery.

Accordingly, the aim of this review is to map how CFs are represented in the physiotherapy literature indexed under that terminology, and to identify where empirical evidence in musculoskeletal rehabilitation is present and where it is absent. The review addresses the following primary question: what evidence on the influence of CFs on pain perception and rehabilitation outcomes in patients with musculoskeletal disorders is available in this literature, and of what kind? The distribution of evidence across direct clinical, indirect mechanistic, and conceptual sources is treated as a finding of the review rather than as an incidental limitation.

## 2. Materials and Methods

This study was conducted as a narrative review with a systematic search strategy, aimed at critically synthesizing the literature on the role of CFs in pain perception and rehabilitation outcomes among individuals with musculoskeletal disorders. This hybrid approach was chosen deliberately, and its rationale is as follows. The conceptual heterogeneity of CFs and the diversity of eligible study designs preclude the pooled synthesis that would justify a systematic review, while transparency of retrieval remains desirable. The search documentation, the selection flow diagram, and the design-appropriate appraisal of empirical studies are therefore retained. The manuscript does not claim completeness of retrieval and is not presented as a systematic review; the reporting of the synthesis was guided by SANRA.

The PRISMA 2020 flow diagram is used solely to document the retrieval and selection process; no PRISMA checklist was completed, no protocol was registered, and the review does not claim the completeness of retrieval that a systematic review requires.

The search was deliberately anchored in the umbrella terms “contextual factors” and “placebo effect”. The aim was not to retrieve all the evidence on the individual constructs under that label, each of which has its own literature, but to characterize the body of physiotherapy literature that adopts the contextual-factors framing. The boundaries of the set are therefore terminological, and the findings are reported as such. 

**Figure 1:**
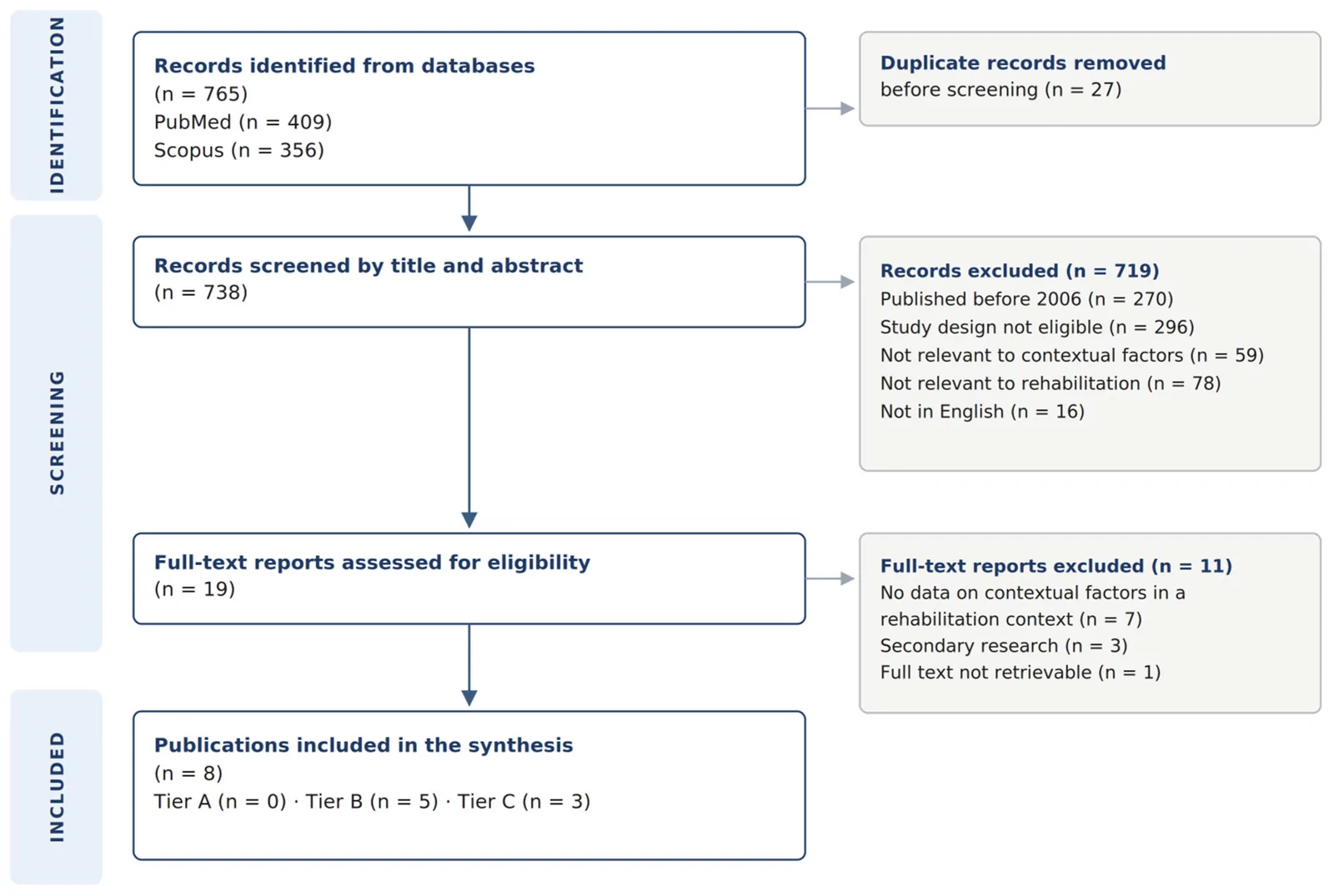
PRISMA 2020 flow diagram of study selection. Duplicate removal, title and abstract screening, and full-text assessment are reported as separate stages, with reasons for exclusion at each stage. The template is used to document the selection process only; the review is reported according to SANRA and is not a systematic review. Adapted from Page MJ et al. [30] (CC BY 4.0).

The authors searched PubMed and Scopus on 31 March 2026. No date or language limits were applied within the databases; publication date and language were handled as eligibility criteria and assessed at title and abstract screening, which is why records published before 2006 and records not written in English appear among the exclusions reported in Figure 1.

The search strategy combined controlled vocabulary and free-text terms related to pain, rehabilitation, and CFs to maximize sensitivity. Full database-specific search strings are reported in Table 1.

For the purposes of screening, CFs were operationally defined, following the international consensus definition of Cook et al. [13] and the domain structure of Testa and Rossettini [12], as elements of the therapeutic encounter that are not attributable to the specific active ingredient of the intervention and that may influence patient expectations, perceptions, or outcomes. Publications addressing such an element without naming it as a CF were eligible provided the element fell within one of those domains.

The search strategy was designed to capture studies investigating the influence of contextual elements on pain perception, treatment response, and rehabilitation outcomes within musculoskeletal settings.

The search yielded 765 records overall, including 409 records retrieved from PubMed and 356 from Scopus. The study identification and selection process is summarized in Figure 1.

Publications were considered eligible if they: (1) investigated CFs and their influence on pain perception or rehabilitation outcomes; (2) concerned musculoskeletal rehabilitation settings or populations, or, where outside that scope, provided mechanistic evidence directly relevant to the review question; (3) involved human participants; (4) were published in English between 2006 and 2026; and (5) provided either original empirical evidence or an explicit conceptual contribution to the definition or structure of CFs in rehabilitation. Criterion 5 was deliberately broadened relative to the original protocol: the conceptual literature is central to how CFs are defined in this field, and excluding it would have misrepresented the state of the discourse. Publications eligible under the conceptual arm of criterion 5, and those eligible under the second clause of criterion 2, are identified as such throughout and are never presented as direct clinical evidence.

Exclusion criteria included studies published before 2006, studies not written in English, studies not addressing CFs, studies unrelated to rehabilitation, duplicate records, and secondary research articles, including systematic reviews and meta-analyses.

Study selection was conducted through sequential title, abstract, and full-text screening. Two reviewers independently assessed all retrieved records for eligibility. Any disagreements were resolved through discussion and consensus, with reference to the predefined inclusion and exclusion criteria.

The three stages of selection are reported separately here and in Figure 1. Twenty-seven duplicate records were removed before screening, leaving 738 records. Of these, 719 were excluded at title and abstract for the following reasons: published before 2006 (n = 270), study design not meeting eligibility criteria (n = 296), non-English language (n = 16), not relevant to contextual factors (n = 59), and not relevant to rehabilitation (n = 78).

Nineteen full texts were assessed for eligibility, of which eleven were excluded: seven did not report data relevant to CFs in a rehabilitation context, three were secondary research, and one was not retrievable in full text. Eight publications were therefore included (Table 2).

Each included publication was classified into one of three evidence tiers, applied consistently in Table 2, in the Results, and in the Discussion. Tier A denotes direct clinical evidence obtained in musculoskeletal rehabilitation populations. Tier B denotes indirect evidence, comprising mechanistic studies in healthy volunteers or in non-musculoskeletal populations, and surveys of clinicians rather than of patients. Tier C denotes conceptual contributions, including perspective articles, which inform interpretation but are never treated as evidence of clinical effect.

To enhance methodological transparency and rigour, the organisation, reporting and interpretation of the results were guided by the Scale for the Assessment of Narrative Review Articles (SANRA) [28].

Although critical appraisal is not mandatory in narrative reviews, methodological quality was evaluated to improve transparency and support interpretation of the available evidence.

Quality assessment was performed according to study design. Randomized controlled trials were assessed using the revised Cochrane Risk of Bias tool (RoB 2) [35], which evaluates five domains of potential bias: (a) bias arising from the randomization process; (b) bias due to deviations from intended interventions; (c) bias due to missing outcome data; (d) bias in outcome measurement; (e) bias in selection of the reported results. In accordance with the RoB 2 guidance, the tool assesses the risk of bias in a specified result rather than in a trial. For the randomized trial retrieved, the results of interest were defined a priori as the between-group (group × time) comparisons from baseline to the immediate post-intervention assessment, for each outcome construct reported. Each result received an overall judgement of low risk of bias, some concerns, or high risk of bias.

Observational and cross-sectional studies were assessed using the Newcastle–Ottawa Scale adapted for cross-sectional studies proposed by Herzog et al. [36], which evaluates methodological quality across three domains: participant selection, comparability of study groups, and outcome assessment. The instrument comprises seven items across three domains: selection (four items, maximum five stars), comparability (one item, maximum two stars), and outcome (two items, maximum three stars), for a maximum of ten stars. Total scores were classified as very good (9–10), good (7–8), satisfactory (5–6), or unsatisfactory (0–4). A star was awarded only where the corresponding methodological feature was explicitly reported; absence of reporting was scored as absence of the feature. Item-level judgements for each study, from which every total score can be reconstructed, are reported in Supplementary Table S1.

Experimental laboratory studies in healthy volunteers using induced-pain paradigms were not appraised with either instrument, since RoB 2 or the adapted NOS. The single study of this type [34] was therefore appraised narratively: it used a controlled between-group design but relied on deception, an unblinded experimenter, no prospective registration, and short-term induced-pain outcomes. It is treated as indirect (Tier B) mechanistic evidence only.

Perspective and conceptual publications were not formally appraised. No validated critical-appraisal instrument applies to this type of literature and appraising it alongside randomized and observational studies would imply an equivalence of evidential status that does not exist. Such publications were used exclusively for conceptual framing.

Quality assessment was independently conducted by two reviewers. Disagreements were resolved through discussion and consensus. The quality appraisal was not used as an exclusion criterion but informed the interpretation of findings and the strength of the conclusions.

Accordingly, findings were synthesized narratively using a thematic approach.

**Figure 2:**
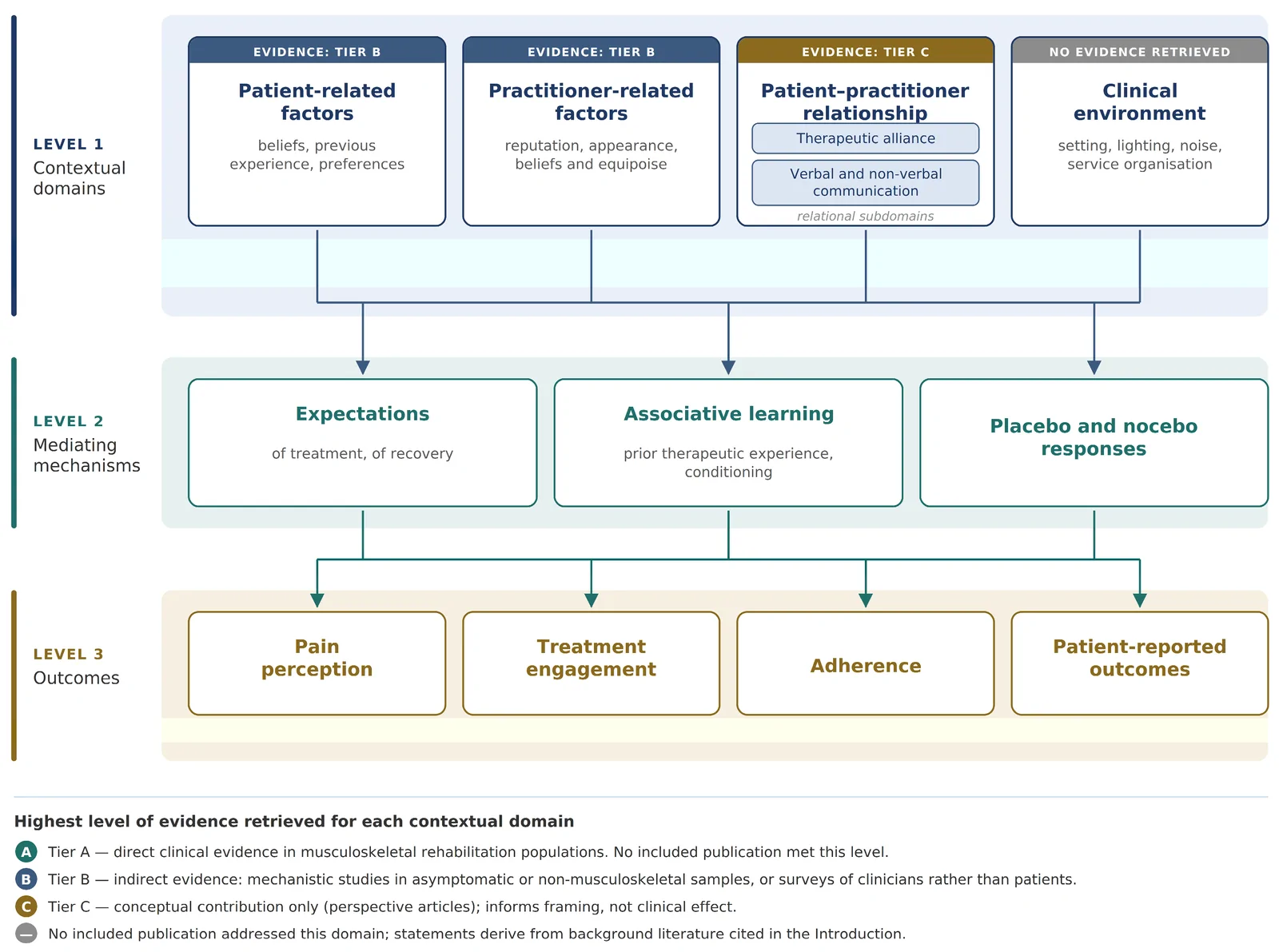
Three-level conceptual model of contextual factors in musculoskeletal rehabilitation. Level 1: Contextual domains; therapeutic alliance and communication are relational subdomains. Level 2: Mechanisms through which domains influence outcomes. Level 3: Outcomes. Header strip: Highest level of evidence for each domain; no domain supported by direct clinical evidence in a musculoskeletal rehabilitation population. This is a conceptual model proposed by the authors, synthesizing frameworks of Testa and Rossettini and of Cook et al. [13], and is not derived empirically from the included publications and has not been empirically validated.

Findings are organized according to a three-level conceptual hierarchy defined a priori and applied consistently throughout the synthesis. The first level comprises contextual domains: patient-related factors, practitioner-related factors, the patient-practitioner relationship, and the clinical environment. Therapeutic alliance and verbal and non-verbal communication are relational subdomains within the third of these, not independent domains. The second level comprises mediating mechanisms through which contextual domains exert their influence: patient expectations, associative learning, and placebo and nocebo responses. The third level comprises outcomes: pain perception, treatment engagement, adherence, and patient-reported measures. This hierarchy is represented graphically in Figure 2 and is used to prevent domains, mechanisms, and outcomes from being treated at the same conceptual level.

Direct clinical evidence in musculoskeletal rehabilitation populations (Tier A) was given primary interpretative weight. Indirect mechanistic evidence (Tier B) was used to support biological plausibility but not to infer clinical effect. Conceptual contributions (Tier C) were used only for framing and are never presented as evidence of effect.

During the preparation of this manuscript, the authors used Claude (Opus 5, Anthropic) to assist with data analysis and interpretation, and with English-language editing and the drafting and formatting of selected portions of text and tables. The authors have reviewed and edited all AI-assisted output and take full responsibility for the content of this publication.

## 3. Results

Eight publications met the eligibility criteria and were included in the narrative synthesis: one randomized controlled trial, two cross-sectional surveys of physiotherapists, one observational study, one experimental study in healthy volunteers, and three perspective articles. Classified by evidence tier, no publication provided direct clinical evidence obtained in a musculoskeletal rehabilitation population (Tier A); five provided indirect mechanistic or implementation-level evidence (Tier B), and three were conceptual contributions (Tier C). The randomized controlled trial retrieved [31] was conducted in asymptomatic volunteers and is therefore classified as Tier B. The absence of Tier A evidence within the literature indexed under this terminology is the principal finding of the present review.

A summary of the characteristics of the studies included is presented in Table 2.

The retrieved literature presents pain and rehabilitation outcomes as emerging from the interaction between contextual domains, the mechanisms through which they operate, and the outcomes they affect, rather than from technical interventions alone. Figure 2 presents the three-level structure adopted in this review as a conceptual model proposed by the authors to organize the synthesis. It is an interpretive framework, not a structure derived from the included publications.

### 3.1. Mediating Mechanisms: Expectations, Placebo and Nocebo Responses

Placebo and nocebo mechanisms were the most frequently addressed contextual domain in the retrieved literature. None of the supporting evidence, however, was obtained in patients with musculoskeletal disorders: it comprises one randomised controlled trial in asymptomatic volunteers, two experimental studies in healthy volunteers or under controlled conditions, and two perspective articles.

Rossettini et al. [22], in a perspective article, propose that positive verbal framing and reassuring communication are associated with reductions in pain intensity and disability while enhancing patients’ expectations regarding treatment efficacy. These findings support the notion that clinician communication may meaningfully influence rehabilitation outcomes beyond the specific therapeutic intervention [22].

Similarly, Safran et al. [30] compared active suboccipital myofascial release with a sham intervention in thirty asymptomatic volunteers, not in patients: individuals with a diagnosed cervical pathology were excluded by design. Within-group improvements were reported for pressure pain threshold, cervical range of motion and proprioception, but no statistically significant group × time interaction was observed for any outcome [31]. Because the trial was not designed or powered as an equivalence study, the absence of a significant interaction does not establish that the two interventions were equivalent, nor does it quantify the contribution of non-specific contextual mechanisms; it is compatible with such a contribution but does not demonstrate one. The trial was conducted in a laboratory setting on asymptomatic participants, measured only immediate post-intervention effects, and was judged to be at high risk of bias for all between-group comparisons (Section 3.5), with a direction of bias that cannot be determined. It therefore constitutes indirect mechanistic evidence and cannot be extrapolated to musculoskeletal rehabilitation populations.

Other studies further reinforced the importance of expectations. Rossettini et al. [11], in a further conceptual contribution, argue that manipulation of expectations modifies pain perception. Likewise, Colloca & Benedetti showed that previous therapeutic experiences conditioned placebo responses through associative learning mechanisms [34].

Although the consistency of these findings strengthens the hypothesis that expectations contribute to pain modulation, most experimental studies were conducted under controlled conditions. Consequently, direct extrapolation to routine rehabilitation settings should be made cautiously.

Poulter et al. [21] also a perspective contribution, advance the argument that clinician beliefs, equipoise, and empathy operate as contextual factors capable of independently shaping pain relief and functional outcomes in musculoskeletal care.

### 3.2. Practitioner-Related Factors: Verbal and Non-Verbal Communication

Evidence regarding non-verbal communication was primarily derived from observational studies. Riquelme et al. showed that facial expressions, posture, and movement behaviors allowed reliable recognition of pain in individuals with cerebral palsy presenting limited verbal communication [33]. Interestingly, parents achieved greater diagnostic accuracy than physiotherapists, highlighting the potential contribution of relational familiarity in interpreting pain-related behaviors.

Overall, although direct evidence linking non-verbal communication to objective rehabilitation outcomes remains limited, available studies consistently indicate that non-verbal interactions represent an important component of the therapeutic context.

These findings concern the recognition of pain-related behaviour in a non-musculoskeletal population and constitute indirect evidence. No included study evaluated whether training clinicians in non-verbal communication alters rehabilitation outcomes, and the present review therefore cannot support claims about the teachability or clinical effect of non-verbal competence.

### 3.3. Implementation of Contextual Factors in Rehabilitation Practice

CFs were also investigated from a broader clinical and organizational perspective. Two national cross-sectional surveys of physiotherapists, rather than of patients, showed that clinicians recognize the clinical importance of CFs [29,32]. Nevertheless, both studies identified a substantial discrepancy between theoretical awareness and practical implementation. Clinicians appeared to underestimate the potential impact of nocebo communication while expressing a strong interest in receiving formal education on contextual mechanisms. The two surveys converge in direction but are not quantitatively comparable: they applied different thresholds when reporting associations between respondent characteristics and answers, so the strength of the associations they report cannot be set side by side. Descriptive proportions are not reproduced here, since one of the two surveys reports discrepant values for the same item in its abstract and in the corresponding table. Because these studies sample clinicians rather than patients, they inform implementation but not clinical effect.

Collectively, these studies suggest that CFs remain insufficiently integrated into routine rehabilitation despite increasing scientific recognition.

Although positive contextual effects were often acknowledged, the potential for nocebo responses was mostly underestimated. Interestingly, almost all respondents expressed a strong interest in formal training on CFs, highlighting a clear discrepancy between awareness and practical competence [32].

Other theoretical contributions have highlighted the limitations of current pain models, emphasising the need for a more comprehensive integration of psychosocial and environmental factors [21].

### 3.4. The Patient–Practitioner Relationship: Therapeutic Alliance

Therapeutic alliance was the domain for which the retrieved literature was most consistent in emphasis, but also the one with the weakest empirical support: no included publication measured or manipulated it directly in patients with musculoskeletal disorders.

Poulter et al. [21] described expectations as being continuously shaped by the quality of the therapeutic relationship rather than solely by the technical characteristics of treatment.

Although direct experimental evidence remains limited, these findings were supported by conceptual models emphasizing dialogue, empathy, shared decision-making, and relational synchrony as central mechanisms through which CFs may influence rehabilitation outcomes.

Taken together and recognizing that no publication retrieved by this review tested the therapeutic alliance experimentally in a musculoskeletal population, the available literature suggests that therapeutic alliance is unlikely to represent an isolated contextual factor. Rather, it appears to function as the relational framework through which several contextual mechanisms—including communication, expectations, and environmental cues—interact during rehabilitation.

### 3.5. Quality of the Studies

The randomized controlled trial was evaluated using the Cochrane Risk of Bias 2 tool (Table 3), and the non-randomized studies using an adapted Newcastle–Ottawa Scale (Table 4). Perspective articles were not appraised, for the reasons given below.

Two design-appropriate instruments were applied. The single randomized controlled trial [31] was assessed with RoB 2. It was judged to be at low risk of bias for the randomization process and for missing outcome data, to raise some concerns for deviations from intended interventions, and to be at high risk of bias for measurement of the outcome and for selection of the reported result. The outcome assessor was not blinded and was also the therapist who delivered both the active and the sham intervention, while all outcomes were operator-dependent; the trial was registered retrospectively, no primary outcome was designated among ten measured variables, and effect sizes were reported for a subset of results only. The overall judgement is therefore high risk of bias. It should be noted that both high-risk domains would be expected to bias results towards the hypothesis of superiority of the active intervention, and no between-group difference was observed; the null result is therefore relatively robust to those two domains. It cannot, however, be read as evidence of equivalence, since the trial was not powered for that purpose. The three non-randomized studies were assessed with the version of the Newcastle–Ottawa Scale adapted for cross-sectional studies, which comprises seven items across three domains for a maximum of ten stars. Scores were 7/10 [29], 6/10 [33], and 5/10 [32], corresponding to good quality for one study and satisfactory quality for the other two. The characterization of non-respondents was absent in all three studies, and comparability was only partially addressed in each; the statistical analyses were appropriate throughout. Reliance on self-report limited the outcome domain in both surveys. One survey additionally showed internal reporting inconsistencies, including a discrepancy between the abstract and the corresponding table for the same item, and reported no ethics approval; these did not affect its score but temper the confidence that can be placed in the figures it reports. Item-level judgements, from which every total score can be reconstructed, are reported in Supplementary Table S1.

The single experimental laboratory study included [34] was appraised narratively rather than with either instrument, for the reasons given in the Methods; its methodological features—deception by design, an unblinded experimenter, absence of prospective registration, and short-term experimentally induced pain outcomes—support its classification as indirect (Tier B) mechanistic evidence only. Perspective and conceptual publications were not formally appraised. No validated instrument exists for the critical appraisal of the conceptual literature and applying one developed for that purpose alongside RoB 2 and the Newcastle–Ottawa Scale would imply an equivalence of evidential status that does not exist. These publications are therefore used exclusively for conceptual framing and are never treated as evidence of clinical effect.

## 4. Discussion and Clinical Implications

The role of CFs in musculoskeletal pain is still the subject of active debate. Clinicians value any factor that improves outcomes, whereas clinical trialists seek to minimize contextual effects to preserve treatment specificity. This phenomenon is therefore framed either as a therapeutic resource or as a source of bias. This issue is further complicated by a methodological disagreement regarding quantification: rigorous estimation would necessitate three-arm randomized controlled trials, which are frequently impractical in rehabilitation. Conversely, alternative methods, such as calculating the proportion attributable to contextual effects, may lead to an overestimation of their impact [21]. These unresolved issues account for the divergent conclusions in the recent literature, cautioning against treating the evidence as definitive.

The conceptual hierarchy adopted in this review—contextual domains, mediating mechanisms, and outcomes—is defined in Section 2 and applied consistently throughout the synthesis. Within that hierarchy, therapeutic alliance is treated as a relational subdomain rather than a distinct category, and placebo and nocebo responses as mediators rather than independent constructs.

The absence of Tier A evidence is itself the most substantive finding of this review. A literature that has produced perspective articles, clinician surveys and laboratory experiments over nearly two decades has not yet produced a single study, within the terminology it uses to define itself, that examines a contextual factor as the variable of interest in patients undergoing musculoskeletal rehabilitation. This is a gap in the evidence base rather than a shortcoming of the present synthesis, and it identifies the priority for future work. The interpretations that follow should be read against that background.

Within those limits, the retrieved literature suggests that CFs, including patient expectations, clinician communication, therapeutic alliance, and environmental characteristics, may contribute to the rehabilitation process by shaping how treatments are perceived, interpreted, and experienced. However, the available evidence is heterogeneous in both methodological quality and study design, requiring cautious interpretation.

One of the most consistent findings across the included studies concerns the role of patient expectations. Within the retrieved literature, this was the domain with the greatest volume of evidence, although none of it was obtained in patients with musculoskeletal disorders. Rossettini et al. [22], in a perspective article, argued that positive verbal framing is associated with lower pain intensity, reduced disability, and improved treatment expectations among individuals with musculoskeletal disorders; that publication does not report original data. Similarly, Safran et al. [31] observed no statistically significant group × time interaction between active and sham interventions; this is compatible with a contribution of non-specific contextual mechanisms but, in a trial neither designed nor powered for equivalence, does not establish one. That trial, however, was conducted in asymptomatic volunteers under laboratory conditions and was judged to be at high risk of bias for all between-group comparisons, and its findings therefore cannot be extrapolated to musculoskeletal rehabilitation populations. Colloca and Benedetti [34] showed experimentally that previous therapeutic experiences condition placebo responses through associative learning; Rossettini et al. [11] advance a similar argument on conceptual grounds.

Collectively, these findings support contemporary neurocognitive models of pain, in which perception is viewed as an active inferential process integrating nociceptive input with previous experiences, expectations, and contextual information. Nevertheless, much of the experimental evidence derives from laboratory settings involving healthy volunteers or highly controlled clinical environments. Consequently, although expectation-related mechanisms appear biologically plausible and clinically relevant, their magnitude and durability within routine rehabilitation practice remain incompletely understood.

Compared with expectation-related mechanisms, evidence regarding non-verbal communication and therapeutic alliance relies predominantly on qualitative and observational research. Riquelme et al. [33] found that facial expressions, posture, and movement behaviours facilitate pain recognition in individuals with limited verbal communication. Likewise, Poulter et al. [21] reported that clinicians’ confidence and interpersonal behaviours influence patients’ expectations and perceived treatment effectiveness.

Although these studies cannot establish causal relationships, their consistency across different methodological approaches suggests that interpersonal interactions represent more than simple adjuncts to rehabilitation.

Rather, communication and therapeutic alliance appear to create the relational context within which rehabilitation interventions are delivered, potentially influencing patient engagement, adherence, self-efficacy, and confidence in recovery. Future longitudinal and interventional studies specifically targeting these relational components are needed to determine whether strengthening therapeutic alliance results in measurable improvements in pain and functional outcomes.

The broader literature on CFs in rehabilitation practice further supports this interpretation. Cross-sectional surveys [29,32] both reported that physiotherapists recognize the importance of CFs but apply them inconsistently during everyday clinical practice. Many clinicians appeared familiar with the potential benefits of positive contextual influences while underestimating the possible negative consequences of nocebo communication. This discrepancy between theoretical knowledge and clinical implementation suggests that contextual competence remains insufficiently addressed during professional education and continuing training.

Importantly, the present review also highlights the multidimensional nature of CFs. Rather than acting independently, expectations, communication, therapeutic alliance, and environmental characteristics appear to interact continuously throughout the rehabilitation process. Positive communication may enhance expectations; effective therapeutic alliance may strengthen treatment adherence; environmental cues may reinforce perceptions of safety or threat. Statements regarding the clinical environment derive from the background literature cited in the Introduction rather than from any publication included in this review; the absence of direct evidence on environmental contextual factors is itself one of the gaps identified within the retrieved set. These reciprocal interactions are consistent with biopsychosocial models of rehabilitation, emphasizing that treatment outcomes emerge from the integration of biological, psychological, interpersonal, and environmental influences rather than from isolated therapeutic techniques.

The findings of this review have several practical implications for rehabilitation professionals managing musculoskeletal pain.

The following considerations are hypotheses generated by the retrieved literature, rather than recommendations supported by it. None are grounded in direct clinical evidence and each requires formal evaluation before they can inform practice.

Firstly, communication may warrant attention as a component of rehabilitation, rather than solely as a means of conveying technical information. Perspective articles argue that providing realistic and non-threatening explanations could reduce nocebo responses. However, none of the included studies tested this theory in patients undergoing musculoskeletal rehabilitation. Secondly, it is suggested that contextual factors extend beyond verbal communication. Non-verbal behaviours have been proposed as a means of shaping perceived empathy and therapeutic support. However, the only study that addressed non-verbal communication examined the recognition of pain-related behaviour in a non-musculoskeletal population, and it does not speak to rehabilitation outcomes. Thirdly, the conceptual literature presents therapeutic alliance as central to patient-centred rehabilitation, yet no included publication has evaluated whether strengthening it alters pain or function. Fourthly, the discrepancy between clinicians’ reported awareness of contextual factors and their routine application suggests that contextual competence may be insufficiently addressed in professional education. However, no included study tested whether such training improves clinical outcomes.

Taken together, these considerations define a research agenda rather than a set of clinical recommendations.

Overall, CFs are not a substitute for evidence-based interventions, but rather a complementary element that, when consciously and ethically integrated, may plausible optimise the effectiveness of treatment and support more individualised, biopsychosocial, patient-centred models of care.

Despite these consistent observations, this review has several limitations. First, much of the included evidence originates from non-clinical, experimental or conceptual sources. Consequently, many of the findings reflect short-term or surrogate outcomes (e.g., experimentally induced pain or immediate changes in expectation) rather than clinically meaningful long-term rehabilitation endpoints. This limits their direct translation into routine practice. Second, there is still a lack of a standardised, universally accepted definition of contextual factors in the field: the use of different terminology and operationalisation across studies reduces comparability and constrains the strength of any synthesis. Third, much of the evidence originates from controlled experimental conditions or specific populations, which restricts the generalisability and external validity of the findings to broader musculoskeletal rehabilitation settings. Fourth, and most importantly, the search strings were anchored on “contextual factors” and “placebo effect” as umbrella terms and did not include therapeutic alliance, empathy, expectations, shared decision-making, clinician communication, or clinical environment as search concepts. The retrieved set therefore reflects the literature explicitly labelled as CFs within physiotherapy and cannot be assumed to represent the empirical evidence on those constructs, much of which is indexed under other terminology. Fifth, the search was restricted to PubMed and Scopus and to English-language publications. A construct-level search aligned with the consensus taxonomy of Cook et al. [13], covering additional rehabilitation databases, is required before the evidence on these constructs can be synthesized. Finally, the heterogeneity of the included designs and the predominantly observational or indirect nature of the evidence preclude firm causal inference. These constraints should be considered when evaluating the clinical and research implications discussed above.

Taken together, the available evidence suggests that CFs should not be regarded as non-specific or incidental aspects of rehabilitation. Instead, they appear to represent integral components of patient-centred care that may influence the effectiveness of rehabilitation interventions by modifying patients’ cognitive, emotional, and behavioural responses. Nevertheless, further high-quality randomized and pragmatic clinical studies are required to clarify the independent contribution of individual contextual domains and to identify effective strategies for their systematic integration into routine rehabilitation practice.

## 5. Conclusions

The central message of this review is dual. CFs have substantial conceptual support and are backed by indirect evidence from mechanistic experiments and implementation-level surveys; direct clinical evidence in musculoskeletal rehabilitation, however, remains lacking. The effectiveness of rehabilitation is therefore unlikely to depend on technical skills alone, but the contribution of CFs to pain perception and rehabilitation outcomes, through cognitive, emotional, interpersonal, and environmental mechanisms, is at present proposed rather than demonstrated.

Within the literature retrieved, no publication studied patients with musculoskeletal disorders in a rehabilitation setting with a contextual factor as the variable of interest. This absence of direct clinical evidence is the principal finding of the review. The proposition that patient expectations, clinician communication, therapeutic alliance and environmental characteristics influence how rehabilitation interventions are perceived and experienced therefore rests, at present, on indirect and conceptual sources. Among the contextual domains identified, expectation-related mechanisms were addressed most frequently, but the supporting evidence consists of experimental work in healthy volunteers, a randomized trial in asymptomatic participants at high risk of bias, and conceptual contributions; therapeutic alliance, non-verbal communication, and environmental influences were supported only by indirect evidence, surveys of clinicians, or conceptual sources. This absence refers to the literature retrieved by the present search strategy and should not be read as evidence that such studies are absent from the wider rehabilitation literature: empirical work on these constructs exists but is largely indexed under construct-specific terminology outside the search concepts applied here.

Rather than replacing evidence-based rehabilitation interventions, CFs should be considered complementary components of patient-centred care that may plausibly optimize the effectiveness of established therapeutic strategies when applied consciously and ethically.

Standardized definitions of CFs are still lacking. Future research should prioritize well-designed pragmatic randomized controlled trials, longitudinal studies, and standardized outcome measures to clarify the independent contribution of CFs and to support their integration into routine rehabilitation practice.

Ultimately, a greater understanding of contextual processes may contribute to more personalized, ethically grounded, and patient-centred rehabilitation while informing future clinical research and professional education.

## Figures and Tables

**Table 1:** Full database-specific search strings.

Database	Search String
PubMed	(“pain” OR “chronic pain” OR “musculoskeletal pain” OR “neuropathic pain”) AND (“Placebo Effect” OR “contextual factors” OR “professional-patient relations” OR “physiotherapist-patient relationship” OR “verbal communication” OR “non-verbal communication”) AND (“Physical Therapy Modalities” OR “physiotherapy” OR “physical therapy”).
Scopus	(“pain” OR “acute pain” OR “chronic pain” OR “musculoskeletal pain” OR “neuropathic pain” OR “psychogenic pain”) AND (“contextual factors” OR “placebo effect” OR “healthcare setting” OR “verbal communication” OR “non-verbal communication” OR “patient-physiotherapist relationship”) AND (“physiotherapy” OR “physical therapy”).

**Table 2:** Characteristics of the studies included in the narrative synthesis.

Study	Design	Evidence Tier	Population	CFs Domain	Main Outcomes	Key Findings
Poulter et al., 2025 [21]	Perspective article	Tier C	MSK pain (clinicians & researchers’ perspective)	Expectations, clinician/patient beliefs, placebo–nocebo	Contextual-effect estimation & clinical translation	Clinician beliefs, equipoise and empathy, and patient expectations, independently modify MSK pain outcomes; real-world/three-arm RCTs with transparent CF reporting advocated.
Safran et al., 2025 [31]	RCT	Tier B	Asymptomatic volunteers (n = 30); cervical pathology an exclusion criterion	Placebo/context effects	Cervical ROM, pain pressure threshold, proprioception	Within-group improvements were reported in both arms; no statistically significant group × time interaction was observed for any outcome. The trial was not designed or powered as an equivalence study.
Rossettini et al., 2022 [22]	Perspective article	Tier C	MSK pain patients	Expectation/placebo-nocebo	Pain, disability	Argues that positive framing and reassuring communication reduce pain intensity and disability; conceptual contribution, no original data reported
Bisconti et al., 2021 [32]	Cross-sectional survey	Tier B	Physiotherapists	Contextual implementation	Knowledge and attitudes	Awareness of CFs was high, whereas knowledge of nocebo mechanisms remained limited
Rossettini et al., 2020 [29]	Cross-sectional survey	Tier B	Physiotherapists	Clinical implementation	Awareness of CFs, professional attitudes	Physiotherapists recognized the importance of contextual factors but reported inconsistent application in routine practice.
Riquelme et al., 2018 [33]	Cross-sectional observational Study	Tier B	Cerebral palsy	Non-verbal communication	Pain recognition	Facial expression, posture, and movement improved recognition of pain-related behaviors.
Rossettini et al., 2018 [11]	Perspective article	Tier C	Not applicable (conceptual)	Expectation	Pain perception	Argues that manipulation of expectations modifies experimentally induced pain; conceptual contribution, no original data reported
Colloca & Benedetti, 2006 [34]	Experimental laboratory study	Tier B	Healthy volunteers with painful stimulus	Learning mechanisms effects	Pain response	Previous therapeutic experiences conditioned subsequent placebo responses.

Tier A, direct clinical evidence in a musculoskeletal rehabilitation population; Tier B, indirect mechanistic or implementation-level evidence; Tier C, conceptual contribution. No included publication met the criteria for Tier A.

**Table 3:** Risk-of-bias assessment of the single randomized controlled trial (RoB 2).

Result Assessed Safran et al., 2025 [31]	D1. Randomization Process ^a^	D2. Deviations from Intended Interventions ^b^	D3. Missing Outcome Data ^c^	D4. Measurement of the Outcome ^d^	D5. Selection of the Reported Result ^e^	Overall
Between-group difference in pressure pain threshold, baseline to immediately post-intervention	Low	Some concerns	Low	High	High	High
Between-group difference in cervical range of motion (flexion, extension, rotation), same time frame	Low	Some concerns	Low	High	High	High
Between-group difference in cervical proprioception (joint repositioning error), same time frame	Low	Some concerns	Low	High	High	High

^a^ Random allocation to two parallel arms of equal size, with comparable baseline characteristics and no imbalance suggesting a problem with the randomization process. ^b^ Participants were blinded to allocation, but the same therapist delivered both the active and the sham intervention while aware of group assignment; adherence and the analysis population are not explicitly described. ^c^ Outcome data available for all randomized participants; no post-randomization attrition reported. ^d^ All outcomes are operator-dependent; the assessor was not blinded to allocation and was also the therapist who delivered both interventions. ^e^ Retrospective registration (NCT06761391, 5 January 2025); no primary outcome designated among the outcome variables measured; effect sizes reported for a subset of comparisons only; no pre-specified analysis plan available.

**Table 4:** Methodological quality of the non-randomized studies, assessed with the Newcastle–Ottawa Scale adapted for cross-sectional studies [36] (maximum ten stars). Quality levels: very good 9–10, good 7–8, satisfactory 5–6, unsatisfactory 0–4. Item-level judgements are reported in Supplementary Table S1.

Study	Selection (Max 5)	Comparability (Max 2)	Outcome (Max 3)	Total Score (Max 10)	Quality Level
Rossettini et al., 2020 [29]	4	1	2	7	Good
Bisconti et al., 2021 [32]	2	1	2	5	Satisfactory
Riquelme et al., 2018 [33]	2	1	3	6	Satisfactory

## Data Availability

No new data were created or analyzed in this study. Data sharing is not applicable to this article.

## Supplementary Materials

The following supporting information can be downloaded at: https://www.mdpi.com/article/10.3390/jcm15166489/s1, Table S1: Item-level methodological quality assessment of the non-randomized studies using the Newcastle-Ottawa Scale adapted for cross-sectional studies.

## Abbreviations

The following abbreviations are used in this manuscript:

CF	Contextual factor
IASP	International Association for the Study of Pain
MSK	Musculoskeletal
PRISMA	Preferred Reporting Items for Systematic Reviews and Meta-Analyses
RCT	Randomized controlled trial
RoB2	Cochrane Risk of Bias 2 tool
ROM	Range of motion
SANRA	Scale for the Assessment of Narrative Review Articles
