# Supplementary material for: More than Rehabilitation: The Role of Contextual Factors in Pain Perception and Recovery: What the Physiotherapy Literature Reports—A Narrative Review with a Systematic Search Strategy
Source: J Clin Med. 2026 Aug 21;15(16):6489. doi: 10.3390/jcm15166489 (PMC13514572; doi:10.3390/jcm15166489)
Supplement: Supplementary file 1 [file jcm-15-06489-s001.zip › jcm-4482019-supplementary.pdf]

Supplementary Table S1. Item-level methodological quality assessment of the non-randomised studies, using the Newcastle–Ottawa Scale adapted for cross-sectional studies.

#	NOS item (cross-sectional adaptation)	Rossettini 2020	Bisconti 2021	Riquelme 2018
S1	Representativeness of the sample (0–1)	1	1	0
S2	Sample size justified and satisfactory (0–1)	1	0	0
S3	Non-respondents described / response rate reported (0–1)	0	0	0
S4	Ascertainment of the exposure or measurement instrument (0–2)	2	1	2
C1	Comparability: control for the principal confounder and for any additional confounder (0–2)	1	1	1
O1	Assessment of the outcome (0–2)	1	1	2
O2	Statistical test appropriate and adequately described (0–1)	1	1	1
Selection subtotal (max 5)		4	2	2
Comparability subtotal (max 2)		1	1	1
Outcome subtotal (max 3)		2	2	3
Total score (max 10)		7	5	6
Quality level		Good	Satisfactory	Satisfactory
